# The association between SYNTAX score and long-term outcomes in patients with unstable angina pectoris: a single-centre retrospective study

**DOI:** 10.1186/s12872-022-02604-x

**Published:** 2022-04-07

**Authors:** Min Xu, Hui Chen, Hong-Wei Li

**Affiliations:** 1 Department of Cardiology, Beijing Friendship Hospital, Capital Medical University, No. 95, Rd. Yong’an, XiCheng District, Beijing, 100050 China; 2 Department of Geriatrics, Beijing Friendship Hospital, Capital Medical University, Beijing, China

**Keywords:** Coronary heart disease, Unstable angina pectoris, SYNTAX score, Major adverse cardiovascular events

## Abstract

**Background:**

The SYNTAX score affects clinical outcomes in early studies. However, the prognostic value of the SYNTAX Score for long-term outcomes and differences by SYNTAX score risk stratification in long-term prognosis between medical therapy and percutaneous coronary intervention (PCI) in patients with unstable angina pectoris (UAP) are not well known in the era of new generation drug-eluting stents and medication.

**Methods:**

In this single-centre retrospective study, a total of 2364 patients with UAP from January 2014 to June 2017 at Beijing Friendship Hospital were enrolled. The primary endpoint was a composite of major adverse cardiovascular events (MACEs), including all-cause death, cardiac death, nonfatal myocardial infarction and stroke at least 2 years after discharge.

**Results:**

In this study, 1695 patients had low SYNTAX scores ($\leqq 22$), 432 patients had medium SYNTAX scores (23–32), 237 patients had high SYNTAX scores (≥ 33), 1018 received medical therapy, and 1346 patients underwent PCI. Long-term MACEs occurred in 95 patients during the 3.38 ± 0.99-year follow-up. Compared to the medical therapy group, the PCI group showed lower MACEs and cardiac death in patients with high SYNTAX scores (7.4% vs. 16.7%, *P* = 0.048; 3.7% vs. 14.6%, *P* = 0.004) but no reduction in patients with low and medium SYNTAX scores. Cox multivariate regression analysis showed that advanced age, diabetes mellitus, left ventricular ejection fraction (LVEF), hs-CRP and high SYNTAX score were independent predictors for MACEs in the medical therapy group (*P* < 0.05), whereas chronic kidney disease (CKD) and LVEF were predictors of MACEs in the PCI group.

**Conclusions:**

Compared to medical therapy, PCI could only significantly reduce long-term MACEs and cardiac death for patients with high SYNTAX scores but not for patients with low and medium SYNTAX scores. A high SYNTAX score could predict long-term MACEs for UAP patients in the medical therapy group but not in the PCI group.

## Introduction

In recent decades, coronary heart disease (CHD) has remained a major cause of mortality worldwide, especially in developing countries. The Synergy between Percutaneous Coronary Intervention with TAXus and Cardiac Surgery (SYNTAX) score is a tool to measure the complexity of coronary lesions and has been recommended for risk stratification and treatment decision-making for untreated left main trunk or three-vessel CHD [1, 2]. Previous studies confirmed that a higher SYNTAX score was related to a worse short-term and long-term prognosis of patients with CHD [3–7]. However, in the era of new generation drug-eluting stents and advanced medicine treatment, it is unclear whether the SYNTAX score can predict long-term MACEs in CHD patients undergoing medical therapy or PCI.

Moreover, although revascularization can improve symptoms in CHD patients with middle-high risk stratification in early studies, most risk scoring systems, such as GRACE and TIMI score, included clinical indicators, without considering characteristics of coronary artery disease [8–10]. Using the SYNTAX score to assess differences in long-term outcomes between medical therapy and PCI has seldom been found. Therefore, we investigated the long-term outcomes in patients with UAP who underwent medical therapy or PCI.

## Method

### Population

The present research was a single-centre, retrospective, observational study. Data came from the UAP patient database of the Cardiovascular Center Beijing Friendship Hospital Database Bank (CBD-BANK), which included UAP patients treated from January 2014 to June 2017 in the Department of Cardiology, Beijing Friendship Hospital. All eligible patients were more than 18 years old and had symptoms of angina as well as at least ≥ 50% luminal stenosis in vessels ≥ 1.5 mm confirmed by coronary angiography. UAP was diagnosed according to the criteria of the European Society of Cardiology guidelines [11]. Patients were excluded if they had previous PCI or coronary artery bypass grafting (CABG), which could affect the accuracy of the SYNTAX score. Severe anaemia, coronary artery bridge, coronary artery spasm, malignant tumour and incomplete information were also exclusion criteria.

The treatment strategy was decided by the physician according to coronary angiography. Coronary flow reserve fraction (FFR), optical coherence tomography (OCT) or intravascular ultrasound (IVUS) were performed in borderline coronary lesions to decide the treatment. If coronary artery diameter stenosis was less than 75% in UAP patients, or coronary artery function examination or endovascular imaging was negative, medical therapy was given; otherwise, PCI was performed. Drug-eluting stents included Xience V (Abbott Vascular, USA), Endeavor Resolute (Medtronic Vascular, USA), Excel (JW Medical Systems, China), Firebird II and Firehawk (MicroPort Medical, China).

All patients were given secondary prevention medicine during hospitalization and after discharge and were followed up regularly by outpatient or telephone. According to the final treatment method, patients were divided into two groups: the medical therapy group and the PCI group. This research was performed according to guidelines set by the Helsinki Declaration and was approved by the ethics commission of the institutional review board of Beijing Friendship Hospital.

### Data collection

The following data were retrospectively collected from the CBD-BANK: (1) demographic factors and cardiovascular risk factors, including sex, age, family history of CHD, smoking, hypertension, diabetes mellitus, dyslipidaemia, stroke, peripheral arterial disease (PAD), and heart failure; and (2) laboratory data at admission, including haemoglobin, fasting plasma glucose (FPG), serum lipids, serum creatinine, body mass index (BMI), and LVEF. (3) Calculation of the SYNTAX score was performed as follows: Using the SYNTAX score calculator (available at http://www.syntaxscore.com), two experienced interventional cardiologists retrospectively calculated the SYNTAX score according to the diagnostic angiograms obtained prior to PCI. The total score was calculated by adding up all individual scores for each separate lesion with a stenosis diameter ≥ 50% in a vessel ≥ 1.5 mm in diameter by visual assessment. SYNTAX scores were categorized as low SYNTAX scores (≤ 22), medium SYNTAX scores (23–32) and high SYNTAX scores (≥ 33).

### Study definitions

Hypertension was defined as having a previous diagnosis of hypertension or being diagnosed during hospitalization. Diabetes mellitus was defined as having a previous diagnosis of diabetes mellitus or being diagnosed during hospitalization. Dyslipidaemia was defined as having a history of hyperlipidaemia, or total cholesterol (TC) ≥ 5.2 mmol/L, low-density lipoprotein cholesterol (LDL-C) ≥ 3.4 mmol/L, triglyceride (TG) ≥ 1.7 mmol/L, or high-density lipoprotein cholesterol (HDL-C) < 1.0 mmol/L on admission. Hyperuricaemia was defined as a serum uric acid concentration > 420 µmol/L in males and > 360 µmol/L in females [12]. Patients who used to smoke or currently smoke were considered to be smokers. The family history of CHD was any immediate family member (parents, siblings) having CHD. Stroke was defined as a ≥ 24 h ischaemic or haemorrhagic cerebrovascular event confirmed by a neurologist. Peripheral arterial disease was defined as having a previous diagnosis of peripheral arterial disease (including carotid artery, subclavian artery, lower extremity arterial disease). Heart failure (HF) was defined as a previous diagnosis of HF or LVEF < 50% on admission. Chronic kidney disease (CKD) was defined as an eGFR < 60 mL/min/1.73 m^2^. The estimated glomerular filtration rate (eGFR) was calculated based on the Chronic Kidney Disease Epidemiology Collaboration creatinine equation [13]. BMI was determined by dividing the patients’ weight in kilograms by the square of the patients’ height in metres.

### The endpoint

The primary endpoint was a composite of MACEs, which included all-cause death, cardiac death, nonfatal myocardial infarction (MI) and stroke at least 2 years after discharge. The secondary endpoints were all-cause death and cardiac death. Comparisons of MACEs between medical therapy and the PCI at each SYNTAX score risk stratification were performed. Predictors of long-term MACEs were also analysed.

### Statistical analysis

Continuous data, expressed as the mean ± SD, and comparisons between 2 groups were analysed using unpaired Student's *t* test or Wilcoxon test, and comparisons of continuous variables among multiple groups were analysed using 1-way analysis of variance. Categorical data, expressed by numbers and percentages, were compared using the chi-square test or Fisher's exact test in cases with cell values < 5. The Kaplan–Meier method was adopted to estimate long-term outcomes among multiple groups and compared by the log-rank test. Cox proportional hazards regression was used to identify MACEs predictors, and all variables with a *P* < 0.1 in the univariate analysis were used to carry out the multivariate analysis. A two-sided *P* < 0.05 was considered to be statistically significant. SPSS version 17.0 (SPSS, Chicago, IL, USA) was applied to conduct all analyses.

## Results

**Fig. 1:**
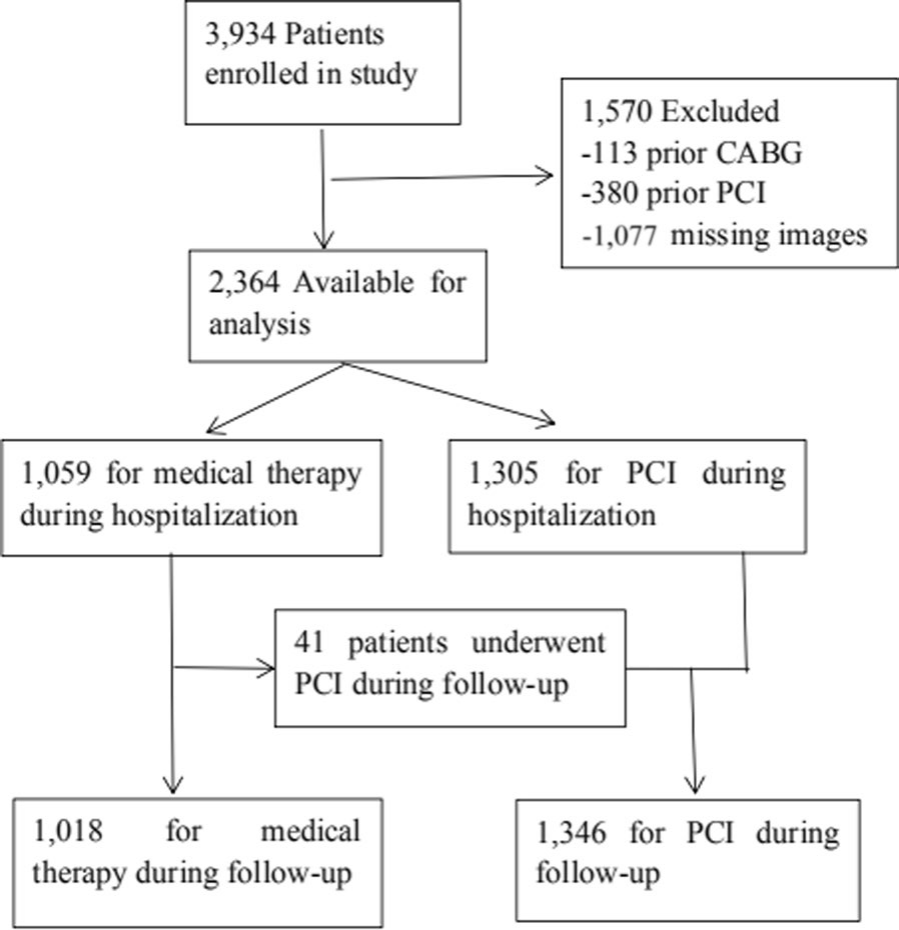
Study design

Overall, 3934 patients with UAP were included in this study, of which 1570 patients were excluded, including 113 with prior CABG, 380 with prior PCI and 1077 patients with missing data. Finally, 2364 patients were enrolled (Fig. 1), including 1432 (60.6%) men and 932 (39.4%) women. The mean age was 64.92 ± 9.84 years (range 28–90). In this study, 1059 patients received medical therapy during hospitalization, of which 41 patients underwent PCI in the follow-up period. Finally, 1018 (43.1%) patients underwent medical therapy, and 1346 (56.9%) patients underwent PCI (Fig. 1).

### Differences in baseline characteristics among SYNTAX score groups

In this study, the mean SYNTAX score was 17.53 ± 10.61 (range 2–66), 1695 (71.7%) patients had low SYNTAX scores, 432 (18.3%) patients had medium SYNTAX scores, and 237 (10%) patients had high SYNTAX scores. The baseline characteristics according to SYNTAX score risk stratification are presented in Table 1. The mean age and prevalence of smoking, diabetes, previous MI, heart failure, and the mean levels of FPG, LDL-C and hs-CRP were significantly higher in patients with medium and high SYNTAX scores than in those with low SYNTAX scores (all *P* < 0.05). Compared to patients with low SYNTAX scores, patients with medium and high SYNTAX scores had lower LVEF level (*P* < 0.001). There were no differences in hypertension, family history of CHD, prior stroke, PAD, atrial fibrillation, or the mean level of heart rate, BMI, TG, TC and serum uric acid among patients with low-medium- and high-SYNTAX scores (all *P* > 0.05). No differences in baseline clinical characteristics existed between patients with medium SYNTAX scores and those with high SYNTAX scores, apart from lower LVEF and more male patients with high SYNTAX scores (*P* < 0.05, Table 1).

**Table 1:** Baseline characteristics by SYNTAX score

	SYNTAX score ≤ 22 (N = 1695)	SYNTAX score 23–32 (N = 432)	SYNTAX score ≥ 33 (N = 237)	*P* value
Age in years (mean, SD)	64.50 ± 9.72	65.79 ± 9.99^a^	66.34 ± 10.20^a^	< *0.05*
Male, n (%)	989 (58.3)	273 (63.2)	170 (71.7)^a,b^	< *0.001*
History of smoking, n (%)	779 (46.0)	226 (52.3)^a^	135 (57.0)^a^	< *0.05*
Hypertension, n (%)	1225 (72.3)	325 (75.2)	171 (72.2)	0.454
Diabetes mellitus, n (%)	571 (33.7)	190 (44.0)^a^	115 (48.5)^a^	< *0.001*
Family history of CHD, n (%)	455 (26.8)	117 (27.1)	53 (22.4)	0.323
Previous MI, n (%)	104 (6.1)	53 (12.3)^a^	33 (13.9)^a^	< *0.001*
Atrial fibrillation, n (%)	98 (5.8)	22 (5.1)	8 (3.4)	0.293
Chronic kidney disease, n (%)	164 (9.7)	60 (13.9)	39 (16.5)	< *0.05*
Prior stroke, n (%)	265 (15.6)	73 (16.9)	45 (19.0)	0.385
Prior PAD, n (%)	129 (7.6)	34 (7.9)	27 (11.4)	0.132
Heart failure, n (%)	77 (4.5)	33 (7.6)^a^	23 (9.7^)a^	< *0.05*
LVEF (%)	67 ± 7	65 ± 9^a^	64 ± 8^a,b^	< *0.001*
BMI (kg/m^2^)	25.93 ± 3.51	25.77 ± 3.53	25.74 ± 3.48	0.563
FPG (mmol/L)	5.73 ± 1.65	6.25 ± 2.27^a^	6.53 ± 2.14^a^	< *0.001*
Triglyceride (mmol/L)	1.63 ± 1.15	1.65 ± 0.99	1.65 ± 1.13	0.897
Total cholesterol (mmol/L)	4.29 ± 1.02	4.41 ± 1.14	4.42 ± 1.11	0.050
HDL-C (mmol/L)	1.15 ± 0.28	1.12 ± 0.27	1.12 ± 0.25	0.069
LDL-C (mmol/L)	2.42 ± 0.74	2.51 ± 0.81^a^	2.54 ± 0.79^a^	< *0.05*
Serum uric acid (umol/L)	339.56 ± 82.94	342.54 ± 83.10	345.81 ± 88.26	0.430
hs-CRP (mg/L)	3.26 ± 5.46	4.07 ± 6.72^a^	4.79 6.83^a^	< *0.001*

*CHD* Coronary heart disease, *MI* myocardial infarction, *FPG* fasting plasma glucose, *PAD* peripheral arterial disease, *LVEF* left ventricular ejection fraction, *BMI* body mass index, *HDL-C* low density lipoprotein cholesterol-C, *LDL-C* low density lipoprotein cholesterol-C, *hs-CRP* high-sensitivity C-reactive protein, Italic values indicate statistical significance

^a^Compare with low SYNTAX score

^b^COMPARE with medium SYNTAX score

### PCI versus medical therapy in baseline characteristics

Compared to patients in the medical therapy group, patients in the PCI group had more cardiovascular risk factors and comorbidities: the frequencies of male patients, smoking, diabetes, previous MI and the levels of FPG, TG, TC, LDL-C, and serum uric acid were notably higher (all *P* < 0.05). In contrast, the mean age, atrial fibrillation and the level of LVEF and HDL-C in the PCI group were lower than those in the medical therapy group (all *P* < 0.05). There were no differences in hypertension, family history of CHD, prior stroke, PAD, heart failure, CKD, or the mean level of BMI and hs-CRP between the PCI group and medical therapy group (all *P* > 0.05). The PCI group had a higher incidence of medium- and high-SYNTAX scores than the medical therapy group (26.1% vs. 8.0%; 14.0% vs. 4.7%, *P* < 0.05, Table 2).

**Table 2:** PCI versus medical therapy in baseline characteristics

	Medical therapy group (n = 1018)	PCI group (n = 1346)	*P* value
Age in years	65.6 ± 9.6	64.4 ± 10.0	< *0.05*
Male, n (%)	562 (55.2)	870 (64.6)	< *0.001*
History of smoking, n (%)	442 (43.4)	698 (51.9)	< *0.001*
Hypertension, n (%)	769 (75.5)	1004 (74.6)	0.598
Diabetes mellitus, n (%)	368 (36.1)	573 (42.6)	< *0.05*
Family history of CHD, n (%)	270 (26.5)	355 (26.4)	0.936
Previous MI, n (%)	65 (6.4)	125 (9.3)	< *0.05*
Atrial fibrillation, n (%)	73 (7.2)	55 (4.1)	< 0.05
Chronic kidney disease, n (%)	114 (11.2)	149 (11.1)	0.992
Prior stroke, n (%)	153 (15.0)	230 (17.1)	0.179
PAD, n (%)	77 (7.6)	113 (8.4)	0.462
Heart failure, n (%)	55 (5.4)	78 (5.8)	0.682
LVEF (%)	67 ± 7	66 ± 8	< *0.05*
Heart rate (bpm)	70.6 ± 11.2	70.8 ± 11.6	0.663
BMI (kg/m^2^)	25.84 ± 3.57	25.92 ± 3.47	0.577
FPG (mmol/L)	7.85 ± 3.18	8.38 ± 3.94	< *0.05*
Triglyceride (mmol/L)	1.56 ± 1.16	1.68 ± 1.09	< *0.05*
Total cholesterol (mmol/L)	4.27 ± 0.96	4.37 ± 1.11	< *0.05*
HDL-C (mmol/L)	1.17 ± 0.27	1.12 ± 0.27	< *0.001*
LDL-C (mmol/L)	2.39 ± 0.70	2.49 ± 0.79	< *0.05*
Serum uric acid (umol/L)	335 ± 85	345 ± 82	< *0.05*
hs-CRP (mg/L)	3.41 ± 5.78	3.67 ± 5.94	0.277
SYNTAX score	12.90 ± 9.33	21.04 ± 10.16	< *0.001*
Low SYNTAX score, n (%)	889 (87.3)	806 (59.9)	< *0.001*
Medium SYNTAX score, n (%)	81 (8.0)	351 (26.1)	
High SYNTAX score, n (%)	48 (4.7)	189 (14.0)	

*MI* myocardial infarction, *FPG* fasting plasma glucose, *PAD* peripheral arterial disease, *LVEF* left ventricular ejection fraction, *BMI* Body mass index, *HDL-C* low density lipoprotein cholesterol-C, *LDL-C* low density lipoprotein cholesterol-C, *PCI* percutaneous coronary intervention, *hs-CRP* high-sensitivity C-reactive protein, Italic values indicate statistical significance

### Comparisons of long-term MACEs by SYNTAX score and treatment

All patients were followed up for 2–6 years with an average of 3.38 ± 0.99 years. MACEs occurred in 95 patients (4.0%): 31 patients experienced noncardiac death, 39 patients experienced cardiac death, 9 patients had acute nonfatal myocardial infarction, and 16 patients had stroke.

**Fig. 2:**
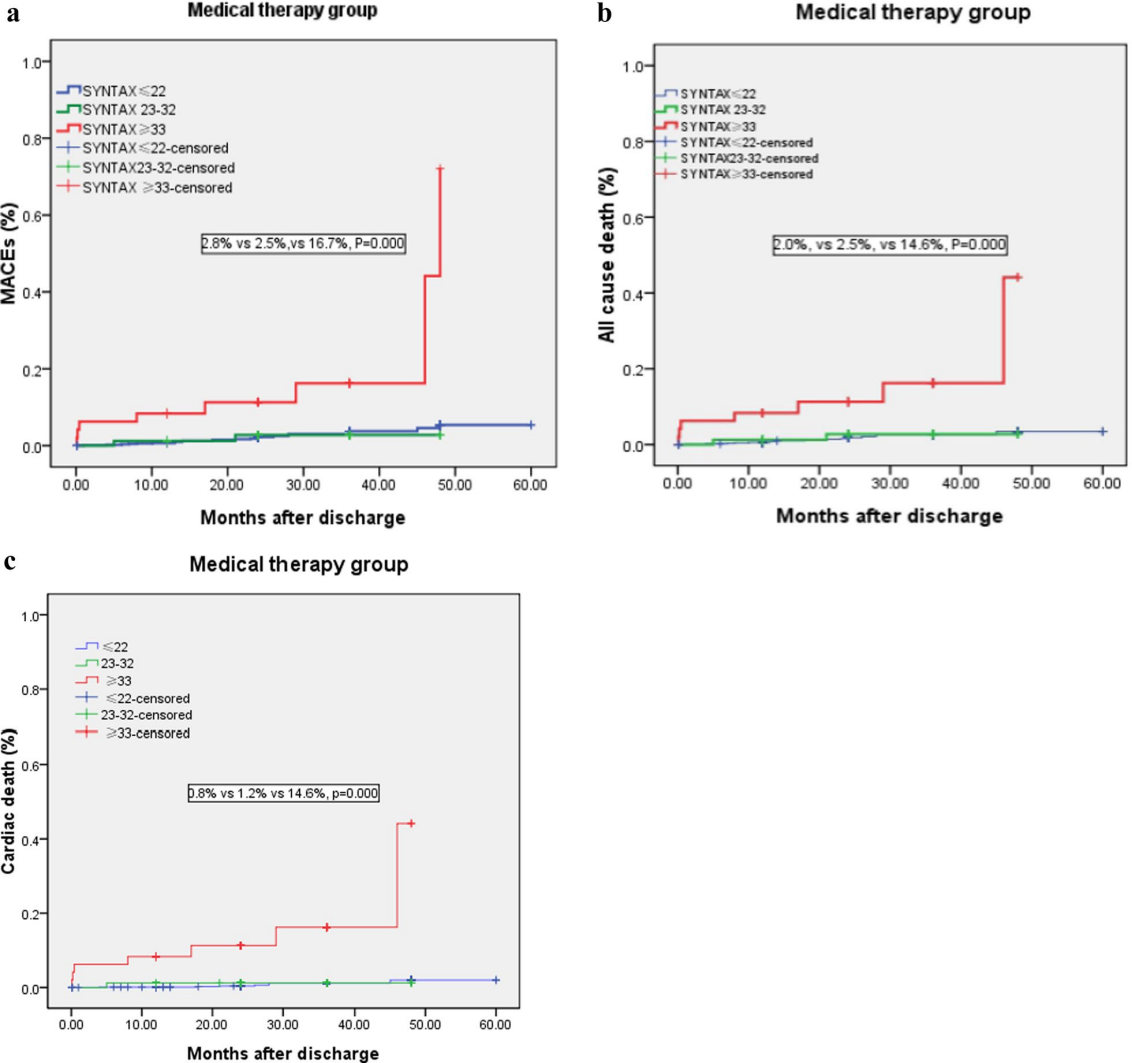
**a** Comparison of MACEs among SYNTAX Score risk stratification in medical therapy group. **b** Comparison of all-cause death among SYNTAX Score risk stratification in medical therapy group. **c** Comparison of cardiac death among SYNTAX Score risk stratification in medical therapy group

In the medical therapy group, the incidence of long-term MACEs in patients with low-, medium- and high-SYNTAX scores was 2.8%, 2.5%, and 16.7%, the all-cause mortality was 2.0%, 2.5%, and 14.6%, and the cardiac mortality was 0.8%, 1.2%, and 14.6%, respectively (Table 3). Kaplan–Meier analysis showed that long-term MACEs, all-cause mortality and cardiac mortality in patients with high SYNTAX scores were higher than those in patients with low and medium SYNTAX scores in the medical therapy group (all *P* < 0.01, Fig. 2a–c).

**Table 3:** PCI versus medical therapy in long-term MACEs according to SYNTAX scores

	Overall			SYNTAX score ≤ 22			SYNTAX score 23–32			SYNTAX score ≥ 33		
	Medical therapy ((n = 1018)	PCI (n = 1346)	*P* value	Medical therapy (n = 889)	PCI (n = 806)	*P* value	Medical therapy (n = 81)	PCI (n = 351)	*P* value	Medical therapy (n = 48)	PCI (n = 189)	*P* value
MACEs, n (%)	35 (3.4)	60 (4.5)	0.211	25 (2.8)	32 (4.0)	0.187	2 (2.5)	14 (4.0)	0.514	8 (16.7)	14 (7.4)	0.048
All-cause death, n (%)	27 (2.7)	43 (3.2)	0.411	18 (2.0)	21 (2.6)	0.426	2 (2.5)	9 (2.6)	0.961	7 (14.6)	13 (6.9)	0.086
Cardiac death, n (%)	13 (1.3)	24(1.8)	0.326	7 (0.8)	13 (1.6)	0.116	1 (1.2)	4 (1.1)	0.943	7 (14.6)	7 (3.7)	0.004
Nonfatal MI, n (%)	3 (0.3)	6 (0.4)	0.555	2 (0.2)	3 (0.4)	0.557	0 (0)	2 (0.6)	0.496	1 (2.1)	1 (0.5)	0.293
Stroke, n (%)	5 (0.5)	11 (0.8)	0.338	5 (0.6)	8 (1.0)	0.311	0 (0)	3 (0.9)	0.404	0 (0)	0 (0)	

*MACE* major adverse cardiovascular events, *MI* myocardial infarction, *PCI* percutaneous coronary intervention, *P* level of statistical significance

**Fig. 3:**
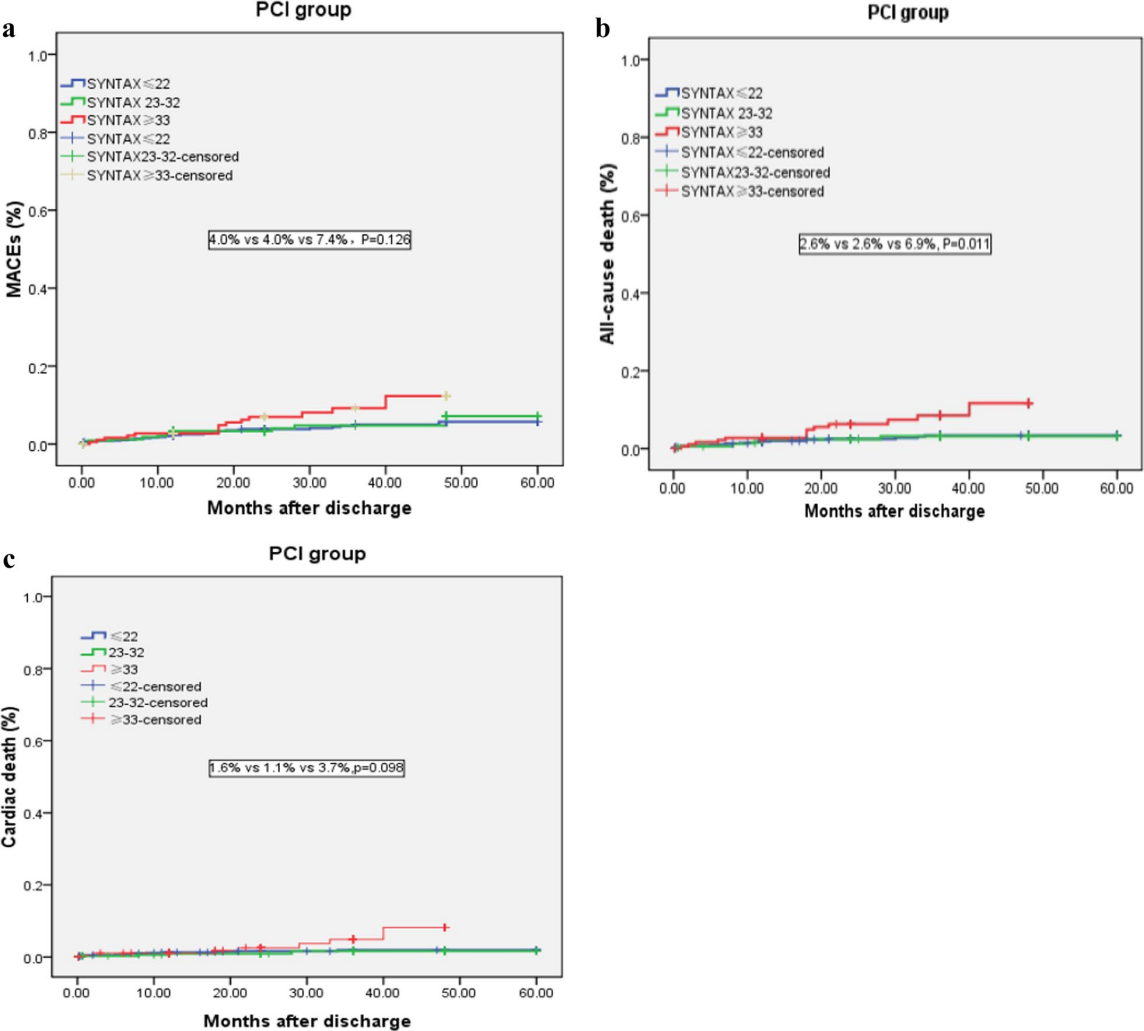
**a** Comparison of MACEs among SYNTAX Score risk stratification in PCI group. **b** Comparison of all-cause death among SYNTAX Score risk stratification in PCI group. **c** Comparison of cardiac death among SYNTAX Score risk stratification in PCI group

In the PCI group, the incidence of long-term MACEs in the low-, medium- and high-SYNTAX groups was 4.0%, 4.0%, and 7.4%, the all-cause mortality was 2.6%, 2.6%, and 6.9%, and the cardiac mortality was 1.6%, 1.1%, and 3.7%, respectively (Table 3). Kaplan–Meier analysis in PCI group was shown in Fig. 3. The results showed that the all-cause mortality in patients with high SYNTAX scores was higher than that in patients with low- and medium-SYNTAX scores (*P* < 0.05, Fig. 3b), while no differences in long-term MACEs and cardiac death were discovered among patients with low-, medium- and high-SYNTAX scores in the PCI group (*P* > 0.05, Fig. 3a, c).

### PCI versus medical therapy in long-term MACEs

The overall MACEs showed no difference between the medical therapy group and the PCI group (*P* > 0.05). According to SYNTAX score risk stratification, there were no differences in long-term MACEs, all-cause mortality, cardiac death, nonfatal MI or stroke between the medical therapy group and the PCI group with low and medium SYNTAX scores (*P* > 0.05). However, in patients with high SYNTAX scores, patients in the medical therapy group showed higher MACEs and cardiac death than patients in the PCI group (*P* < 0.05), whereas no differences in all-cause mortality, nonfatal MI or stroke were detected between the medical therapy group and the PCI group (*P* > 0.05, Table 3).

### Predictors for long-term MACEs in the medical therapy group and the PCI group

According to Cox proportional hazards models with both univariable and multivariable approaches, long-term predictors are shown in Table 4. This result revealed that advanced age (HR 1.096, 95% CI 1.051–1.143; *P* < 0.001), diabetes mellitus (HR 2.873, 95% CI 1.412–5.846; *P* = 0.004), LVEF (HR 0.017, 95% CI 0.000–0.645; *P* = 0.028), SYNTAX score ≥ 33 (HR 4.912, 95% CI 2.108–11.449; *P* < 0.001), and hs-CRP (HR 1.046, 95% CI 1.010–1.083; *P* = 0.011) were independent predictors for long-term MACEs in the medical therapy group (*P* < 0.001). However, CKD (HR 2.998, 95% CI 1.666–5.396; *P* < 0.001) and LVEF (HR 0.009, 95% CI 0.001–0.085; *P* < 0.001) were predictors of long-term MACEs in the PCI group, but a high SYNTAX score was not a predictor of long-term MACEs in the PCI group (*P* > 0.05, Table 4). The Hosmer–Lemeshow test verified that the models of both the medical therapy group and the PCI group were effective (all *P* > 0.1, Table 5).

**Table 4:** Predictors for long-term MACEs in patients underwent medical therapy or PCI

	Medication group				PCI group			
	Univariable analysis		Multivariable analysis		Univariable analysis		Multivariable analysis	
	HR (95% CI)	*P* value	HR (95% CI)	*P* value	HR (95% CI)	*P* value	HR (95% CI)	*P* value
Age in years	1.086 (1.037–61.136)	0.000	1.096 (1.051–1.143)	< *0.001*	1.005 (0.975–1.036)	0.749	–	–
Male	1.338 (0.549–3.266)	0.522	–	–	1.483 (0.725–3.033)	0.280	–	–
History of smoking	0.797 (0.314–2.026)	0.634	–	–	0.776(0.410–1.466)	0.434	–	–
Hypertension	0.883 (0.333–2.338)	0.802	–	–	1.225 (0.652–2.302)	0.529	–	–
Diabetes mellitus	3.181 (1.518–6.668)	0.002	2.873 (1.412–5.846)	0.004	0.916 (0.529–1.584)	0.753	–	–
Hyperlipidemia	0.954 (0.461–1.973)	0.899	–	–	0.697 (0.390–1.246)	0.223	–	–
Hyperuricemia	1.907 (0.683–5.327)	0.218	–	–	1.094 (0.563–2.126)	0792	–	–
Family history of CHD	1.341 (0.4556–3.236)	0.514	–	–	1.271 (0.682–2.369)	0.451	–	–
Previous MI	1.237 (0.320–4.777)	0.758	–	–	0.774 (0.344–1.742)	0.537	–	–
Atrial fibrillation	0.561 (0.190–1.655)	0.295	–	–	0.477 (0.178–1.274)	0.140	–	–
Chronic kidney disease	1.848 (0.810–4.216)	0.145	–		2.751 (1.418–5.334)	0.003	2.998 (1.666–5.396)	< *0.001*
Prior stroke	0.917 (0.358–2.353)	0.857	–	–	1.824 (0.983–3.384)	0.0757	–	–
PAD	0.829 (0.270–2.548)	0.744	–	–	1.429 (0.504–4.053)	0.502	–	–
LVEF	0.010 (0.000–0.766)	0.000	0.017 (0.000–0.645)	0.028	0.023 (0.002–0.344)	0.006	0.009 (0.001–0.085)	< *0.001*
SYNTAX ≥ 33	4.895 (1.840–13.017)	0.001	4.912 (2.108–11.449)	< *0.001*	1.666 (0.889–3.119)	0.111	–	–
BMI	0.966 (0.868–1.075)	0.523	–	–	0.950 (0.875–1.032)	0.226	–	–
Heart rate	1.010 (0.984–1.038)	0.457	–	–	0.992 (0.970–1.015)	0.490	–	–
hs-CRP	1.033 (0.997–1.071)	0.075	1.046 (1.010–1.083)	0.011	1.018 (0.985–1.052)	0.289	–	–

*CHD* coronary heart disease, *MI* Myocardial infarction, *FPG* fasting plasma glucose, *PAD* peripheral arterial disease, *LVEF* left ventricular ejection fraction, *BMI* body mass index, *PCI* percutaneous coronary intervention, *hs-CRP*: high-sensitivity C-reactive protein, Italic values indicate statistical significance

**Table 5:** Hosmer–Lemeshow test for the model of predictors for long-term MACEs

	Chi-square	*df*	*P* value
Medication group	12.527	8	0.129
PCI group	8.426	7	0.297

*PCI* Percutaneous coronary intervention, *P* level of statistical significance

## Discussion

In this study, the main observations were as follows: (1) male sex, advanced age, smoking, diabetes, heart failure, CKD, FPG, LDL-C and hs-CRP were positively and LVEF was negatively correlated with the SYNTAX score; (2) compared to the medical therapy group, PCI could significantly decrease long-term MACEs in patients with high SYNTAX scores but not reduce long-term MACEs in patients with low and medium SYNTAX scores; and (3) a high SYNTAX score was a predictor for long-term MACEs in the medical therapy group but not in the PCI group.

A series of studies have shown that ageing, male sex, diabetes mellitus and impaired renal function were independent predictors of a high SYNTAX score [14–17]. Karadeniz showed that increased hs-CRP was one of the strong predictors of high SYNTAX scores in ACS patients [18]. Minamisawa reported that a high SYNTAX score was associated with heart failure [3]. In this study, we reached the same conclusion. Moreover, we also found that the cardiovascular risk factors for patients with medium SYNTAX scores were similar to those of patients with high SYNTAX scores. Hence, intensive management of cardiovascular risk factors is important. Kaya et al. reported that the SYNTAX score was associated with abdominal aortic intima-media thickness in non-ST elevation myocardial infarction (NSTEMI) [19]. In this study, we found that the prevalence of heart failure and CKD was higher in the mid-high SYNTAX-score groups. This result indicated that a high SYNTAX score may be related to systemic atherosclerosis.

A FRISC-II invasive study confirmed that an early invasive treatment strategy leads to a sustained reduction in mortality, repeat hospital admissions and revascularization in short-term and long-term follow-up periods in unstable coronary artery disease patients, and an early invasive treatment strategy was most effective in patients at higher risk [20]. The SYNTAX trial showed that CABG was better than PCI in the long-term prognosis of CHD patients with high SYNTAX scores [1, 2]. Recently, the BARI-2D trial demonstrated that among patients with diabetes and stable ischaemic heart disease, 5-year MACEs were not lower after PCI than after medical therapy in patients with a low- or mid/high SYNTAX score (17.8% vs. 19.2%, *P* = 0.84; 35.6% vs. 26.5%, *P* = 0.12); however, CABG could reduce MACEs in patients with mid/high SYNTAX scores (15.3% vs. 30.3%, *P* = 0.02) [21]. Our study showed that patients with high SYNTAX scores benefited more from PCI than patients with low and medium SYNTAX scores. This result was inconsistent with previous reports, which may be related to different research populations, as only UAP patients were included in this study.

Studies on SYNTAX scores reported its capacity to predict adverse events for patients undergoing PCI, and the prognostic value of the SYNTAX score in all-cause mortality was also shown at different points in time up to 5 years after PCI [4–7]. Brown AJ reported that increasing the SYNTAX score was an independent predictor of MACEs (HR: 1.61, 95% CI 1.05–2.47, *P* = 0.03) [5]. Eickhoff reported that the SYNTAX score independently predicted 1-year and 2-year mortality in < 75-year-old patients (HR: 1.43, 95% CI 1.03–2.00, *P* = 0.034; and HR: 1.33, 95% CI 1.01–1.76, *P* = 0.041) [6]. A pilot study confirmed that a high SYNTAX score was associated with a 6.2-fold hazard of in-hospital death (OR 6.2, 95% CI 2.6–14.1, *P* < 0.001) and was an independent prognostic marker of in-hospital outcomes in patients with ST elevation myocardial infarction (STEMI) [22]. Our data showed that advanced age, diabetes and high SYNTAX score (≥ 33) were independent predictors of long-term MACEs for UAP patients in the medical therapy group but not in the PCI group. This result indicated that patients with high-risk stratification benefit better from PCI.

Hayıroğlu et al. reported that acute kidney injury (AKI) was an independent predictor of long-term mortality in patients with STEMI complicated by cardiogenic shock who were treated with primary PCI [23]. In our study, CKD was an independent predictor of long-term MACEs in the PCI group but not in the medical therapy group, and the reason may be related to acute kidney injury during PCI. Regrettably, our data did not record the AKI situation, and we will explore this issue in the future. These findings suggest that intensive management of UAP patients with CKD should be strengthened.

## Conclusions

First, male sex, advanced age, smoking, diabetes, heart failure, CKD, FPG, LDL-C and hs-CRP were positively and LVEF was negatively correlated with the SYNTAX score. Second, PCI could significantly reduce long-term MACEs and cardiac death in patients with high SYNTAX scores compared with medical therapy. Third, a high SYNTAX score was a predictor for long-term MACEs in the medical therapy group but not in the PCI group.

### Study limitations

In our study, there were several limitations. First, this was a single-centre, retrospective, observational study, and the results were less convincing than those of randomized controlled trials. Second, UAP patients who received medical therapy or PCI were enrolled, and patients undergoing CABG were not enrolled, so our findings may not be representative of these patients. Third, the SYNTAX score is only a risk stratification of anatomic features of coronary lesions and does not consider the degree of coronary stenosis and clinical factors, so patients with the same SYNTAX score may have different degrees of coronary stenosis, and the prognosis may be different. Fourth, our data did not record AKI and SYNTAX II, which may affect the analysis of the conclusion. Finally, we focused on hard cardiac events in this study, so revascularization was not assessed as an outcome. Future studies should be carried out to examine these details.

## Data Availability

The data used and/or analysed during the current study are available from the corresponding author on reasonable request.

## Abbreviations

CHD: Coronary heart disease
UAP: Unstable angina pectoris
MI: Myocardial infarction
BMI: Body mass index
HDL-C: Low-density lipoprotein cholesterol-C
LDL-C: Low-density lipoprotein cholesterol-C
eGFR: Estimated glomerular filtration rate
PCI: Percutaneous coronary intervention
MACEs: Major adverse cardiovascular events
